# Acute Respiratory Distress Syndrome Exacerbated by Inappropriate Use of Mechanical Insufflation-Exsufflation Following Infection in a Patient With Amyotrophic Lateral Sclerosis: A Case Report

**DOI:** 10.7759/cureus.84991

**Published:** 2025-05-28

**Authors:** Taro Kato, Keisuke Yorimoto, Yosuke Ariake, Yuko Shimizu-Motohashi, Takatoshi Hara

**Affiliations:** 1 Department of Physical Rehabilitation, National Center Hospital, National Center of Neurology and Psychiatry, Tokyo, JPN; 2 Department of Informatics, Graduate School of Informatics and Engineering, The University of Electro-Communications, Tokyo, JPN; 3 Department of Child Neurology, National Center Hospital, National Center of Neurology and Psychiatry, Tokyo, JPN; 4 Department of Rehabilitation Medicine, Jikei University School of Medicine, Tokyo, JPN

**Keywords:** acute hypoxemic respiratory failure, acute respiratory distress syndrome [ards], acute respiratory failure with hypoxia, amyotrophic lateral sclerosis – frontotemporal spectrum disorder, community aquired pneumonia, home respiratory rehabilitation, mechanical insufflation-exsufflation, multidisciplinary care approach, tracheostomy ventilation and amyotrophic lateral sclerosis, ventilator associated pneumonia

## Abstract

Mechanical insufflation-exsufflation (MI-E) is widely used to assist airway secretion clearance in patients with neuromuscular disorders such as amyotrophic lateral sclerosis (ALS). While MI-E is generally considered safe when used intermittently for cough augmentation, its prolonged and unsupervised use as a substitute for invasive ventilation is discouraged by current clinical guidelines, including those issued by the American College of Chest Physicians and the American Academy of Neurology.

We report the case of a 53-year-old man with advanced ALS, diagnosed approximately 10 years earlier, who developed acute respiratory distress syndrome (ARDS) exacerbated by inappropriate use of MI-E following a recent respiratory infection. The patient had previously relied on tracheostomy invasive ventilation (TIV) but chose to suspend its use, instead employing MI-E continuously for 62 hours (28,234 cycles), based on prior positive experiences and personal preference.

Upon hospital admission, the patient was diagnosed with mild ARDS, bacterial pneumonia, and influenza B infection. Although the respiratory infection was likely the primary cause of deterioration, MI-E-related pressure changes may have exacerbated pulmonary injury, particularly in the context of acute infection. Rapid improvement in gas exchange and imaging findings within 48 hours of MI-E discontinuation further supports this hypothesis.

We discuss possible mechanisms linking excessive MI-E usage to lung injury, including barotrauma and negative pressure pulmonary edema. We also emphasize the importance of clearly defined device indications, structured caregiver education, and regular clinical supervision in home respiratory care.

To our knowledge, this may be the first reported case of ARDS potentially resulting from the combined effects of infection and inappropriate MI-E application, highlighting the need for multidisciplinary coordination and proper device supervision in managing advanced neuromuscular respiratory failure at home.

## Introduction

Amyotrophic lateral sclerosis (ALS) is a progressive neurodegenerative disease characterized by the degeneration of motor neurons, leading to progressive respiratory insufficiency, impaired secretion clearance, and ultimately respiratory failure [1,2].

Mechanical insufflation-exsufflation (MI-E) is a commonly used technique that simulates a cough by alternating positive and negative airway pressures, thereby facilitating secretion clearance in patients with neuromuscular disorders [3]. MI-E has been shown to play a crucial role in reducing pulmonary complications in these patients [4]. However, it is not intended to replace invasive mechanical ventilation, particularly in cases requiring continuous respiratory support, due to concerns about pressure-related complications and the lack of evidence supporting its efficacy in this role.

Although MI-E is generally considered safe, rare complications such as pneumothorax and hemodynamic instability have been reported, especially when used inappropriately or for prolonged periods [5,6]. There are currently no published reports establishing a direct link between MI-E use and the onset of acute respiratory distress syndrome (ARDS). Nevertheless, the possibility of MI-E contributing to pulmonary complications resembling ventilator-induced lung injury (VILI) has been discussed, particularly in the context of excessive or unsupervised use.

Here, we present a case of ARDS that developed following a respiratory infection in a patient with advanced ALS, in which prolonged and unsupervised use of MI-E may have acted as a contributing factor in exacerbating lung injury.

This case also underscores the importance of structured respiratory care at home, including clear guidance on device limitations, regular clinical supervision, and comprehensive caregiver education, to ensure the safe and effective use of MI-E in patients with severe neuromuscular conditions.

This position is supported by current clinical guidelines, such as those from the American College of Chest Physicians and the American Academy of Neurology, which recommend MI-E only as an adjunctive airway clearance tool and not as a substitute for ventilatory support [7].

## Case presentation

A 53-year-old man with ALS, diagnosed approximately 10 years earlier, had progressed to complete quadriplegia and was being managed with tracheostomy invasive ventilation (TIV) and a gastrostomy tube. In the winter of that year, the patient experienced worsening dyspnea. Given his previous positive experiences with MI-E and based on a strong personal preference, he independently chose to use MI-E continuously for three consecutive days as an alternative to TIV. Although TIV was not formally discontinued under medical supervision, its use was effectively suspended during this period.

During this time, the patient underwent 62 hours of uninterrupted MI-E, totaling 28,234 insufflation-exsufflation cycles (settings: +30 hPa for 3 seconds, -30 hPa for 2 seconds, with a 1-second pause), using the Cough Assist E70 device (Philips Respironics).

Upon admission, despite receiving 9-10 L/min of oxygen via tracheostomy, his oxygen saturation (SpO₂) remained critically low (60-72%), indicating severe hypoxemia. Arterial blood gas analysis showed a PaO₂ of 64.0 mmHg (normal: 80-100 mmHg), PaCO₂ of 74.8 mmHg (normal: 35-45 mmHg), HCO₃⁻ of 26.8 mEq/L (normal: 22-26 mEq/L), and a PaO₂/FiO₂ Ratio (PF ratio) of 213.3. Brain natriuretic peptide (BNP) was 30 pg/mL (normal: <100 pg/mL).

Chest CT revealed bilateral ground-glass opacities and consolidations. Laboratory tests demonstrated an elevated WBC count of 33,000/μL (normal: 4,000-10,000/μL), with 86.4% neutrophils, and a CRP level of 41.9 mg/dL (normal: <0.3 mg/dL). An influenza B antigen test was positive, and sputum cultures identified *Pseudomonas aeruginosa* and *Acinetobacter* species.

**Figure 1:**
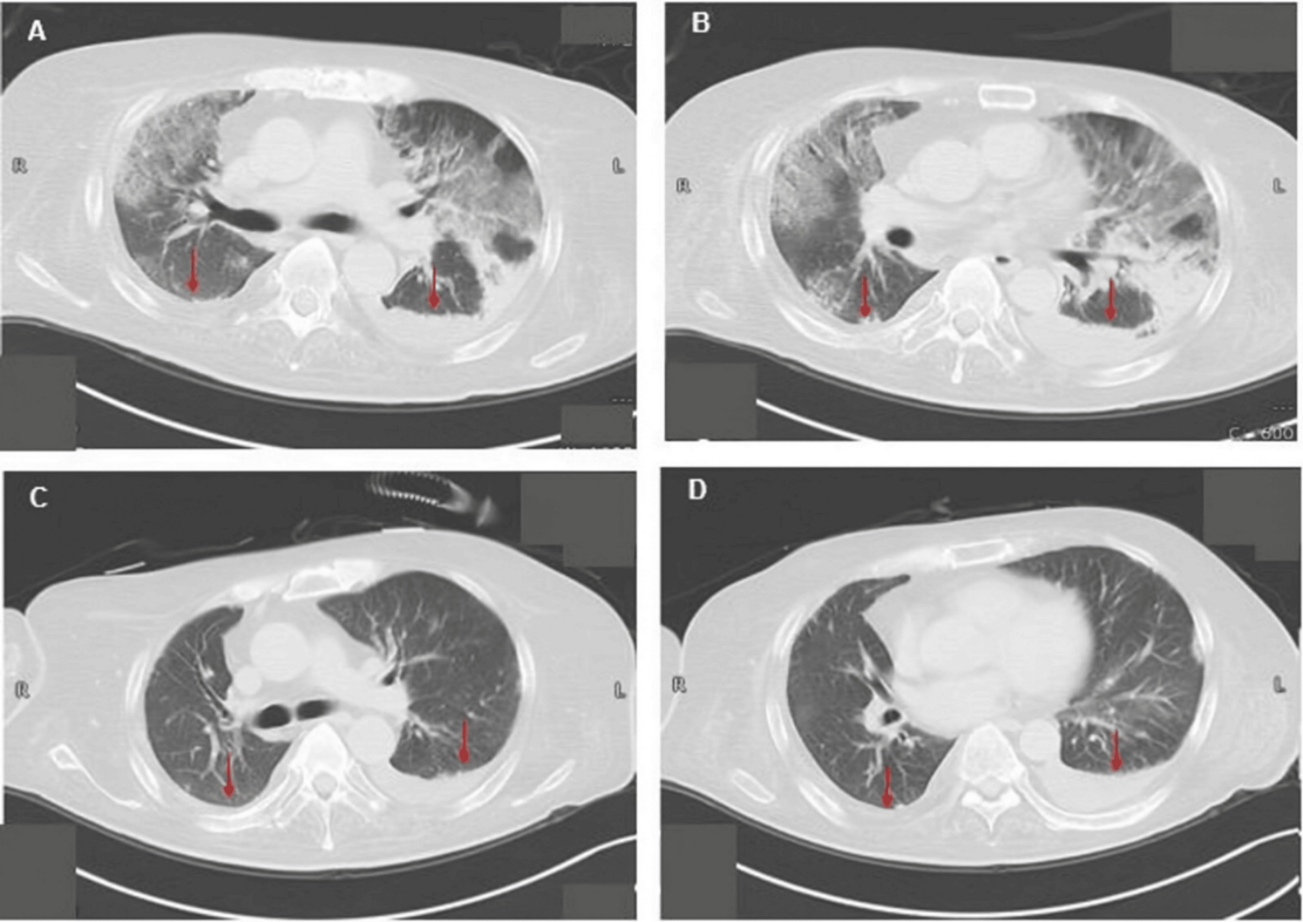
Axial chest CT images on day 0 and day 21. (A) Axial chest CT image obtained on day 0, showing bilateral pulmonary infiltrates predominantly in the posterior regions (arrows). (B) Another axial image from day 0, revealing more extensive bilateral involvement (arrows). (C) Axial chest CT image obtained on day 21, demonstrating marked resolution of infiltrates (arrows). (D) Additional axial CT image from day 21, showing further clearing of pulmonary infiltrates (arrows).

**Figure 2:**
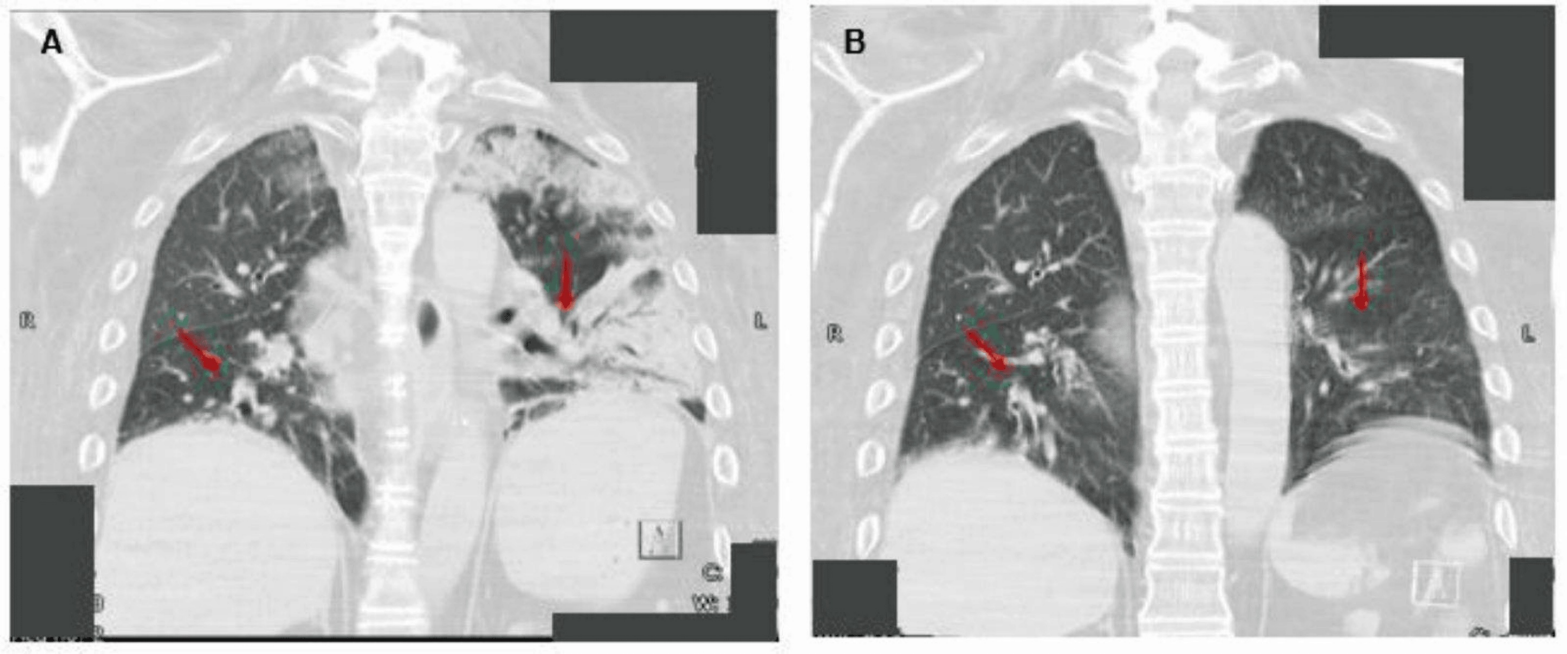
Coronal chest CT images on day 0 and day 21. (A) Coronal CT image on day 0, showing extensive bilateral pulmonary infiltrates, especially in the posterior lower lung fields (arrows). (B) Coronal CT image on day 21, showing clear improvement in the extent and density of bilateral infiltrates (arrows).

Imaging findings are presented in chronological order. Follow-up chest CT performed on Day 21 showed marked improvement in bilateral pulmonary infiltrates (Figures 1-2).

**Figure 3:**
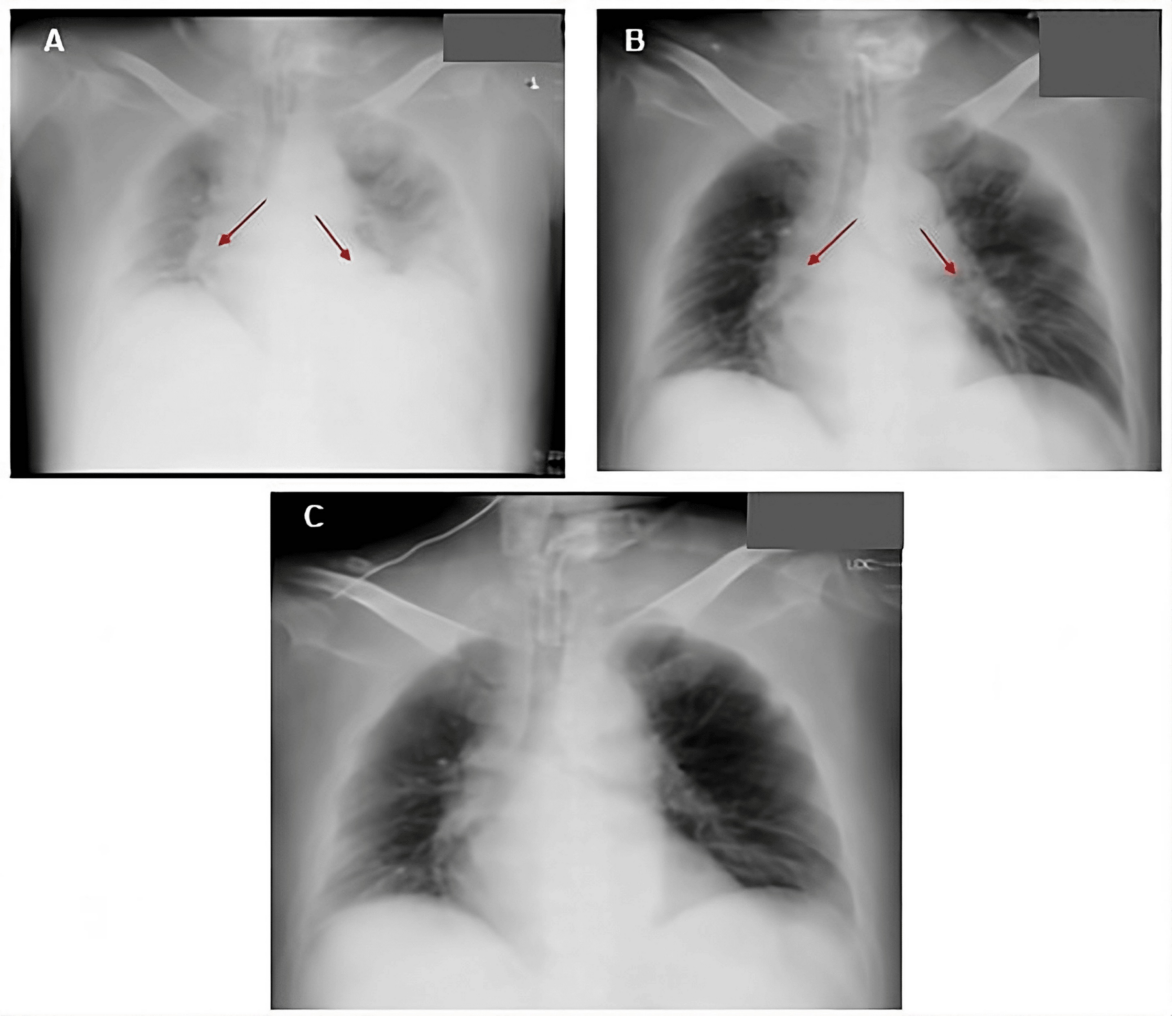
Serial chest radiographs showing progressive resolution of ARDS. (A) Chest radiograph on day 0, showing diffuse bilateral pulmonary opacities consistent with acute respiratory distress syndrome (ARDS) (arrows). (B) Chest radiograph on day 2, showing substantial improvement in pulmonary infiltrates following the discontinuation of continuous mechanical insufflation-exsufflation (MI-E) (arrows). (C) Chest radiograph on day 34, demonstrating near-complete resolution of infiltrates and normalization of lung fields.

Chest radiographs obtained on Day 0, Day 2, and Day 34 confirmed a rapid radiologic improvement within 48 hours of discontinuing MI-E (Figure 3).

Only available imaging was included in the figures; no CT was performed on Day 2.

The patient was diagnosed with mild ARDS, bacterial pneumonia, and influenza B infection. TIV was resumed with adjusted settings (Inspiratory Positive Airway Pressure (IPAP) 24 cmH₂O, Expiratory Positive Airway Pressure (EPAP) 8 cmH₂O, Fraction of Inspired Oxygen (FiO₂) 0.3), and MI-E was modified to an intermittent protocol of five breaths per session, using +40/-40 hPa with 2-second cycles and a 1-second pause, applied throughout the day as needed.

Intravenous tazobactam/piperacillin and peramivir were administered as antibiotic and antiviral therapy, respectively. Both treatments were continued for five days, until clinical and laboratory improvements were achieved. Improvement was observed on Day 2, including increased oxygen saturation, reduced sputum volume, and declining inflammatory markers.

Gas exchange and inflammatory markers improved significantly within 48 hours of MI-E discontinuation. The patient was discharged on Day 36 after extensive caregiver re-education, which included structured guidance on MI-E indications, safe use, and ventilator settings, as well as adjustments to the home respiratory support system.

Table 1 summarizes the temporal changes in arterial blood gases and inflammatory markers before and after discontinuation of continuous MI-E.

**Table 1:** Time course of respiratory and inflammatory parameters before and after cessation of continuous MI-E. MI-E: Mechanical insufflation-exsufflation; PaO₂: Partial pressure of oxygen (normal range: 80-100 mmHg); FiO₂: Fraction of inspired oxygen; PF ratio: PaO₂/FiO₂ ratio (calculated by dividing PaO₂ by FiO₂); PaCO₂: Partial pressure of carbon dioxide (normal range: 35-45 mmHg); CRP: C-reactive protein (normal range: <0.3 mg/dL); WBC = white blood cell count (normal range: 4,000-10,000/μL). Chest imaging findings = radiographic observations of the lungs.

Date	MI-E status	PaO₂ (mmHg)	FiO₂	PF ratio (PaO₂/FiO₂)	PaCO₂ (mmHg)	CRP (mg/dL)	WBC (/μL)	Chest imaging findings
Day 0 (admission)	Continuous use for 62 hours	64	0.3	213	74.8	41.9	33,170	Bilateral diffuse ground-glass opacities and consolidations
Day 2 (48 h after MI-E cessation)	MI-E stopped for 48 hours	100	0.3	333	45.7	20.2	22,050	Marked improvement of infiltrates
Day 34	Continued cessation / limited use	95.8	0.21	456	29.1	1.2	7,700	Complete resolution and normalization

## Discussion

This case highlights the possibility that prolonged and inappropriate continuous use of MI-E, initiated in response to respiratory discomfort following infection, may have contributed to or aggravated the development of ARDS. Following hospitalization, the MI-E settings were modified, and the device was transitioned to regulated, intermittent use under clinical supervision.

Although MI-E is designed to assist with secretion clearance, extended and unsupervised application may pose risks of pulmonary complications resembling ventilator-induced lung injury (VILI) [8]. Repeated application of high positive and negative pressures can result in barotrauma or volutrauma, while abrupt shifts in transpulmonary pressure may promote pulmonary capillary leakage and damage to alveolar structures [9,10].

The negative pressure phase of MI-E may also increase venous return and pulmonary blood volume, potentially leading to hydrostatic pulmonary edema or even pulmonary hemorrhage, particularly in vulnerable patients [11]. In this case, the absence of elevated brain natriuretic peptide (BNP 30 pg/mL) made cardiogenic pulmonary edema unlikely, supporting a non-cardiac etiology.

The patient presented with ARDS, bacterial pneumonia, and influenza B infection, suggesting a multifactorial etiology. While infection and chronic respiratory insufficiency were likely the primary contributors, inappropriate MI-E use may have played a contributing role in the severity of lung injury.

The clinical course showed rapid improvement after discontinuation of continuous MI-E and initiation of intermittent use. Within 48 hours, gas exchange and inflammatory markers improved substantially, as reflected by a drop in PaCO₂ from 74.8 to 45.7 mmHg, an increase in the PaO₂/FiO₂ ratio from 213 to 333, normalization of WBC count, and a marked decrease in CRP from 41.9 mg/dL to near-normal levels.

Chest radiographs obtained on Day 0, Day 2, and Day 34 also demonstrated progressive radiographic improvement, particularly within the first 48 hours after MI-E discontinuation (Figure 3).

Although the respiratory infection likely served as the initial trigger, the additive effects of prolonged MI-E use may have intensified the lung injury, resulting in a clinical picture consistent with ARDS. This interplay highlights the diagnostic complexity of distinguishing infection-induced ARDS from device-related pulmonary complications in patients with ALS.

While a definitive causal relationship between MI-E and ARDS cannot be established in this case, the temporal sequence, pathophysiological plausibility, and rapid improvement following MI-E cessation support a possible contributing role. This underscores the need for close clinical monitoring and proper application of MI-E, particularly in home care settings where caregivers may lack specialized training.

In this case, the patient’s strong insistence on using MI-E, based on previous positive outcomes, created a complex ethical and practical dilemma. Despite signs of clinical deterioration, caregivers were reluctant or unable to override the patient’s preference, revealing a systemic gap in the shared decision-making framework of home respiratory management.

Recent studies have emphasized the importance of structured home respiratory care and caregiver training in reducing complications associated with MI-E use [7,12-13]. Additionally, online educational tools and standardized training programs have been proposed to improve the consistency and safety of MI-E application in home environments [14,15].

These insights highlight the urgent need for clearer clinical guidelines and standardized caregiver education to empower non-specialist caregivers to recognize evolving clinical risks and respond effectively, even when patient preferences conflict with evidence-based recommendations.

Further clinical research and post-market surveillance are warranted to better understand the safety profile of MI-E under various conditions, particularly during concurrent infections, and to inform the development of evidence-based protocols for home-based MI-E use.

## Conclusions

MI-E remains an essential tool for airway secretion clearance in patients with neuromuscular disorders such as ALS. However, it should not be used as a substitute for invasive ventilatory support, particularly in cases requiring continuous respiratory assistance, as current clinical guidelines recommend MI-E only as an adjunctive therapy for airway clearance.

This case illustrates the potential for serious pulmonary complications, including ARDS, when MI-E is used continuously and without adequate medical supervision. The patient’s condition demonstrated marked improvement after transitioning from prolonged, unsupervised use to regulated, intermittent application, underscoring the importance of appropriate usage.

It also highlights the complexity of decision-making in home care settings, where caregivers may feel pressured to honor patient preferences, even when those preferences conflict with evolving clinical risks.

To ensure the safe and effective use of MI-E, clinical supervision, structured caregiver education, and explicit guidance on device limitations are essential components of home-based respiratory care.

Further investigation is warranted to clarify the safety profile of MI-E across diverse clinical scenarios, especially when used during active infection, and to inform the development of evidence-based protocols that guide its safe application in non-hospital settings.
